# Effect of Fish Meal Replacement by Complex Protein on Growth Performance, Metabolism, and Health of Triploid Rainbow Trout (*Oncorhynchus mykiss*)

**DOI:** 10.1155/anu/7379050

**Published:** 2026-05-18

**Authors:** Hongdian Zhu, Dong Huang, Rui Ma, Guoliang Sun, Zezhong Wu, Haining Tian, Yuqiong Meng

**Affiliations:** ^1^ College of Eco-Environmental Engineering, Qinghai University, Xining, 810016, China, qhu.edu.cn; ^2^ Key Laboratory of Plateau Cold-Water Fish Culture and Eco-Environmental Conservation (Co-Construction by Ministry and Province), Ministry of Agriculture and Rural Affairs, Qinghai University, Xining, 810016, China, qhu.edu.cn

**Keywords:** complex protein, fish meal replacement, metabolic health, sustainable aquaculture, triploid rainbow trout

## Abstract

A comprehensive investigation was conducted to evaluate dietary fish meal (FM) replacement by a complex protein blend composed of soybean meal, soybean protein concentrate, corn gluten meal, wheat gluten, and blood cell powder in triploid rainbow trout (*Oncorhynchus mykiss*). Five isonitrogenous (46% crude protein) and isolipidic (24% crude lipid) diets with graded FM substitution levels (0%, 25%, 50%, 75%, and 100%) were fed for 12 weeks. Complete FM replacement did not significantly affect survival, weight gain rate, or feed conversion ratio (*p* > 0.05). However, these growth‐related outcomes occurred together with clear changes in body composition, nutrient metabolism, and health‐related indicators. In particular, complete replacement was associated with elevated alanine aminotransferase (ALT) and aspartate aminotransferase (AST) activities, increased hepatic MDA content, altered digestive and metabolic responses, and changes in intestinal morphology and immune‐related gene expression. These results indicate that complete FM replacement with the tested complex protein blend maintained short‐term growth performance in subadult triploid rainbow trout but also induced clear signs of hepatic stress and intestinal inflammatory activation. Therefore, 100% replacement should not be regarded as a fully validated or risk‐free strategy for aquaculture production, and its suitability should be interpreted cautiously despite the maintenance of growth during the 12‐week trial.

## 1. Introduction

The global aquaculture industry has expanded rapidly in response to increasing demand for aquatic protein [1]. Fish meal (FM) remains a preferred aquafeed ingredient because of its high protein quality, balanced amino acid profile, and high digestibility. However, continued dependence on FM and fish oil has raised concerns regarding sustainability, supply, and cost [2, 3].

To reduce FM use, a wide range of alternative protein sources has been explored in aquafeeds, including plant proteins, animal byproducts, insect meals, and microbial or single‐cell proteins [3–7]. However, reliance on a single alternative protein source often introduces nutritional or functional limitations, such as amino acid imbalance, antinutritional factors, reduced nutrient availability, or lower palatability. In rainbow trout, adverse responses to high inclusion of soybean‐ or rapeseed‐based ingredients have been reported, including impaired growth and intestinal or physiological disturbance [8–11]. These limitations have increased interest in multiprotein systems rather than single‐ingredient replacement strategies.

Combining different protein sources has been proposed as a practical way to improve amino acid complementarity and the overall utility of alternative diets [12–14]. Composite protein systems have shown promising effects in several cultured species, including Pacific white shrimp, Chinese perch, red sea bream, and largemouth bass [15–18]. In rainbow trout, optimized FM‐free plant‐protein mixtures supplemented with essential amino acids and taurine have also supported acceptable growth under certain conditions [19]. Based on this concept, and considering practical feasibility and cost, the present study used a complex protein blend composed of soybean meal, soybean protein concentrate, corn gluten meal, wheat gluten, and blood cell powder. This formulation was derived from an improved commercial feed framework and designed according to the principle of ingredient complementarity rather than reliance on a single alternative protein source. Wheat gluten is recognized as a useful and highly digestible protein ingredient in aquafeeds [20], whereas blood‐derived animal proteins have been used in rainbow trout diets, and rendered animal protein ingredients are generally characterized by high digestibility in this species [21, 22]. Accordingly, the present blend was intended to combine commonly used plant protein ingredients with an animal‐derived protein source to provide a practically relevant and nutritionally complementary alternative to FM.

In rainbow trout, the response to FM replacement is strongly ingredient‐ and formulation‐dependent. Plant‐protein combinations or other mixed alternative‐protein formulations have supported partial or moderate replacement in some studies [19, 23–26]. Animal byproduct mixtures have also produced variable results, ranging from only limited acceptable replacement in juvenile trout [27] to successful complete replacement in one specific mixed‐formulation study [28]. Ingredient‐evaluation work has further shown marked differences among soybean, pea, and canola meals and their concentrates [29], and high dietary inclusion of plant protein mixtures has been associated with altered muscle growth dynamics in rainbow trout [30]. Together, these findings indicate that higher replacement levels require careful formulation, particularly with respect to ingredient selection, amino acid balance, digestibility, and feed acceptance [19, 26, 29]. Nevertheless, studies on complex protein substitution in rainbow trout remain limited, especially in triploid fish.

This point is relevant because the present study focused on triploid rainbow trout, whereas direct diploid–triploid comparisons under complex protein substitution are still lacking. Previous studies in subadult triploid rainbow trout have shown that dietary protein and lipid levels can influence antioxidant capacity, protein digestion and absorption, growth performance, and health status [31, 32]. These findings support the view that triploid trout should be considered within a specific nutritional context. At the same time, because the present experiment did not include a diploid control group, we do not attempt to infer ploidy‐dependent differences. Rather, the present results are intended to describe the response of triploid rainbow trout to the tested FM replacement strategy only. In addition, available trout studies suggest that maintenance of growth does not necessarily exclude nutritionally relevant constraints when ingredient composition is changed substantially [26, 29, 30].

In this context, the present study evaluated the effects of graded FM replacement with a complex protein mixture composed of soybean meal, soybean protein concentrate, corn gluten meal, wheat gluten, and blood cell powder on growth performance, digestion, nutrient transport, metabolism, and health‐related indicators in triploid rainbow trout. The results provide experimental evidence for assessing a practically derived and nutritionally complementary FM replacement strategy in triploid fish. However, because the diet was tested as a combined formulation, the respective effects of soybean meal, soybean protein concentrate, corn gluten meal, wheat gluten, and blood cell powder cannot be separated, and because no diploid group was included, direct ploidy‐based comparisons were not possible.

## 2. Materials and Methods

The present study was approved by the Ethical Committee of Qinghai University. The research protocols were implemented under the standard operating procedures (SOPs) of the Guide for the Use of Experimental Animals of Qinghai University.

### 2.1. Experimental Diets

The specific proportion of soybean meal, soybean protein concentrate, corn gluten meal, wheat gluten, and blood cell powder was based on an improved commercial feed formulation provided by Tongwei Group Co., Ltd., and was adjusted with consideration of formulation feasibility and practical cost.

Based on the optimal protein and lipid levels of triploid rainbow trout feed on the Qinghai‐Tibet Plateau [31, 32], a complex protein blend consisting of soybean meal, soybean protein concentrate, corn gluten meal, wheat gluten, and blood cell powder was formulated to replace FM in varying proportions of 0%, 25%, 50%, 75%, and 100%. The blend was designed to combine commonly used plant protein ingredients with an animal‐derived protein source so as to improve overall protein complementarity and provide a more balanced alternative to FM than any single ingredient alone. Soybean meal and soybean protein concentrate served as the main plant protein components, corn gluten meal and wheat gluten were included to optimize the protein profile of the formulation, and blood cell powder was incorporated as a highly concentrated animal protein ingredient. Accordingly, five groups (100% FM, 25% CP, 50% CP, 75% CP, and 100% CP) of isonitrogenous and isolipidic diets were prepared (Tables 1 and 2). Betaine (0.5%) was included in all diets as a common feed additive; however, no additional fish soluble or fish protein hydrolysates were used in the 100% CP diet. All experimental feeds were processed by Tongwei Group Co., Ltd., China into 4 mm slow‐sinking extruded feeds, and then stored at −20°C for further use.

**Table 1:** Formulation and proximate composition of the experimental diets.

Ingredients	100% FM	25% CP	50% CP	75% CP	100% CP
Fish meal^a^	60.0	45.0	30.0	15.0	0.0
Soybean meal^a^	0.0	5.5	11.0	16.5	22.0
Soy protein concentrate^a^	0.0	2.5	5.0	7.5	10.0
Corn gluten meal^a^	0.00	2.25	4.50	6.75	9.00
Wheat gluten^a^	0.0	2.0	4.0	6.0	8.0
Blood cell powder^a^	0.0	2.5	5.0	7.5	10.0
Wheat meal^a^	13.5	13.5	13.5	13.5	13.5
Cellulose	5.12	3.92	2.57	1.22	0.00
Fish oil	18.60	19.50	20.50	21.50	22.52
Mineral and vitamin premix^b^	1.0	1.0	1.0	1.0	1.0
Ca(H_2_PO_4_)_2_	0.8	0.8	0.8	0.8	0.8
Choline chloride	0.3	0.3	0.3	0.3	0.3
Mold inhibitor	0.1	0.1	0.1	0.1	0.1
Ethoxyquin	0.05	0.05	0.05	0.05	0.05
Betaine	0.5	0.5	0.5	0.5	0.5
Astaxanthin^c^	0.03	0.03	0.03	0.03	0.03
Lysine HCL (75%)	0.0	0.4	0.8	1.2	1.5
DL‐methionine	0.00	0.15	0.35	0.55	0.70
Total	100	100	100	100	100
Proximate analysis					
Moisture (%)	3.96	4.01	4.06	4.65	4.01
Crude protein (% dry matter)	47.48	46.87	46.48	47.54	46.73
Crude lipid (% dry matter)	24.70	24.27	24.40	23.66	23.77
Ash (% dry matter)	11.6	9.14	7.81	6.99	6.74
Energy (KJ/g dry matter)	23.78	24.16	24.67	23.65	23.63
Fatty acids (% of identified fatty acids)					
C18:2n‐6	5.72	5.60	4.56	5.76	5.94
C20:4n‐6	1.38	2.49	2.44	2.40	2.43
C18:3n‐3	4.02	1.54	1.41	1.50	1.57
C20:5n‐3	5.37	9.00	9.19	8.68	8.67
C22:6n‐3	8.07	13.76	13.45	12.54	12.26

^a^Fish meal, crude protein 72%, crude lipid 8.7%; soybean meal, crude protein 53.5%, crude lipid 3.1%; soy protein concentrate, crude protein 70.2%, crude lipid 0.5%; corn gluten meal, crude protein 67.7%, crude lipid 3.1%; wheat gluten, crude protein 84.6%, crude lipid 2.0%; blood cell powder, crude protein 95.2%, crude lipid 1.7%; wheat meal, crude protein 17.6%, crude lipid 1.5%. The complex protein blend was formulated to combine plant protein ingredients with an animal‐derived protein source to improve protein complementarity and provide a practical alternative to fish meal.

^b^Both of these ingredients were supplied by Tongwei Co., Ltd., China. Vitamin premix, vitamin premix for carnivorous fish: 0.5%; mineral premix, mineral premix for carnivorous fish: 0.5%.

^c^Astaxanthin, the effective content is 10% (CAROPHYLL, DSM, Netherlands).

**Table 2:** Amino acid composition of the experimental diets.

Ingredients	100% FM	25% CP	50% CP	75% CP	100% CP
Essential amino acids (% dry matter)					
Lysine	2.97	3.14	3.36	3.27	3.21
Methionine	1.00	1.11	1.24	1.10	1.08
Phenylalanine	1.84	1.97	2.04	2.14	2.12
Threonine	1.47	1.49	1.54	1.48	1.43
Isoleucine	1.82	1.74	1.76	1.76	1.69
Leucine	3.32	3.38	3.62	3.78	3.74
Valine	2.11	2.06	2.08	2.12	2.05
Histidine	1.61	1.82	1.91	2.04	2.14
Arginine	2.13	2.19	2.14	2.07	2.03
Nonessential amino acids (% dry matter)					
Aspartic acid	3.85	3.71	3.73	3.66	3.73
Glycine	2.66	2.48	2.41	2.41	2.34
Alanine	2.68	2.58	2.58	2.61	2.55
Cysteine	0.40	0.51	0.52	0.50	0.60
Tyrosine	1.38	1.34	1.35	1.42	1.35
Proline	2.19	2.31	2.53	2.75	2.78
Glutamic acid	6.17	6.38	6.75	7.12	7.13
Serine	1.73	1.78	1.85	1.94	1.95
Cysteine	0.40	0.51	0.50	0.60	0.60

### 2.2. Feeding Trial

Triploid rainbow trout (*Oncorhynchus mykiss*), with an initial weight of 208 ± 15 g, were obtained from a commercial fish farm in Qinghai, China. These were subadult fish. After 24 h of fasting, 2000 fish were randomly distributed into 20 cages (4 m × 4 m × 6 m) at a density of 100 fish per cage. The 12‐week feeding trial was conducted following a 2‐week acclimation period. During the feeding period, fish were not subjected to chronic starvation or feed restriction; the only fasting procedures were the standard 24 h fast before stocking and the 48 h fast before terminal sampling. It is important to note that the results of this study apply specifically to subadult fish, as juvenile rainbow trout may not tolerate plant protein‐based diets as well as subadults or larger fish. Fish were fed twice daily (08:30 and 16:30) to apparent satiation. During the experiment, feed intake, mortality, water temperature (8–16°C), and dissolved oxygen (>7 mg/L) were monitored daily.

### 2.3. Sample Collection and Calculations

At the termination of the 12‐week feeding trial, all fish underwent a 48 h fasting period prior to biometric assessment. From each experimental cage, 12 randomly selected specimens were euthanized using eugenol anesthetic (1:10,000 dilution; Shanghai Reagent Corp., China) following established ichthyological protocols.

For body composition analysis, three fish per cage were immediately frozen at −20°C. Another three specimens were measured for morphometric parameters. Hematological samples were collected from three additional fish per cage via caudal venipuncture (2 mL) into lithium heparin anticoagulant tubes. Blood samples were centrifuged at 3000 × *g* for 20 min at 4°C to obtain plasma, which was subsequently stored at −80°C until biochemical analysis.

Gastrointestinal tissues (stomach, pyloric ceca, liver, and intestine) were aseptically dissected, partitioned, flash‐frozen in liquid nitrogen, and subsequently maintained at −80°C. For histological examination, representative segments of mid‐intestine (1 cm) and hepatic tissue (1 cm^3^) were fixed in 4% paraformaldehyde. All samples, excluding those designated for histology, were pooled within each cage (*n* = 3 fish) to constitute one biological replicate, yielding four replicates per dietary treatment for subsequent analytical procedures.

Survival %=100×final number of fish/initial number of fish,

Weight gain rate WGR, %=100×final body weight g − initial body weight g/initial body weight g,

Feed conversion ratio FCR=feed fed g/body weight gain g,

Feed intake FI, %/day=100×feed fed g/days×initial body weight g+final body weight g/2,

Protein efficiency ratio PER=body weight gain g/feed fed g×dietary protein level %,

Condition factor CF=100×body weight g/body length cm3,

Viscerosomatic index VSI, %=100×viscera weight g/body weight g,

Hepatosomatic index HSI, %=100×hepatic weight g/body weight g,

Intestinal length index ILI= intestine length mm/body length mm.

### 2.4. Biochemical Analysis

The proximate composition of feed ingredients, experimental diets, and whole fish bodies was analyzed following AOAC (2005) official methods. Sample preparation involved mechanical pulverization to achieve homogeneous particle size prior to analytical procedures. Moisture content was determined gravimetrically by oven‐drying samples at 105°C (Model DHG‐9070A, Jinghong Laboratory Equipment Co., China) until constant weight was attained. Nitrogen content quantification was conducted using the Kjeldahl method (N × 6.25 conversion factor) with an automated analyzer system (Kjeltec 2300, Foss Analytical, Denmark). Lipid content analysis was performed via Soxhlet extraction employing diethyl ether as the solvent (Soxtec 8000, Foss Analytical, Denmark). Ash content determination involved combustion in a muffle furnace (HXM‐2000, Huaxing Furnace Industry, China) maintained at 550°C until complete mineralization and constant weight were achieved.

Amino acid composition in experimental feeds was determined using an automated amino acid analyzer (LA8800, Hitachi, Japan) following established protocols. Lysine, threonine, and arginine were quantified using the acid hydrolysis method described by a previous study [33]. Methionine analysis required oxidative pretreatment involving reaction with performic acid (30% hydrogen peroxide:88% formic acid, 1:9 v/v) at 4°C for 16 h, followed by hydrobromic acid quenching and solvent evaporation using a vacuum concentrator (TVE‐1100 A, EYELA, Japan) prior to acid hydrolysis. All hydrolyzed samples were filtered through 0.22 μm membranes and analyzed using norleucine as an internal standard for quantitative calibration.

Tissue homogenates (10% w/v) were prepared from stomach, pyloric ceca, intestine, and liver samples using ice‐cold physiological saline (1:9 tissue‐to‐buffer ratio) in a refrigerated homogenizer (XHF‐D, Xinzhi, China) at 4°C. Following centrifugation (10,000 × *g*, 15 min, 4°C), the resulting supernatant was aliquoted and stored at −80°C for further analysis. Protease activity was analyzed according to a previous study [34]. Other biochemical parameters were assessed using commercial kits (Nanjing Jiancheng Bioengineering Institute, China) as follows: total protein (TP) (#A045‐2‐2), malondialdehyde (MDA, #A003‐1‐2), total antioxidant capacity (T‐AOC, #A015‐3‐1), lipase (#A054‐1‐1), amylase (#C016‐1‐1), alanine aminotransferase (ALT, #C009‐2‐1), aspartate aminotransferase (AST, #C010‐2‐1), superoxide dismutase (SOD, #A001‐3‐2), catalase (CAT, #A007‐1‐1), and glutathione peroxidase (GSH‐PX, #A005‐1‐2). All assays were performed in strict accordance with the manufacturers’ protocols, with appropriate quality controls and technical replicates to ensure data reliability.

Plasma biochemical parameters were analyzed using an automated biochemical analyzer (ADVIA 2400, SIEMENS, Germany) following standardized clinical protocols. The measured parameters included: ALT, AST, TP, albumin (ALB), globulin (GLOB), alkaline phosphatase (ALP), glucose (GLU), cholesterol (CHOL), triglycerides (TG), high‐density lipoprotein cholesterol (HDL‐C), low‐density lipoprotein cholesterol (LDL‐C), creatine phosphokinase (CPK), and lactate dehydrogenase (LDH).

### 2.5. Liver and Intestinal Morphology Analysis

For histological examination, one representative tissue section per cage (four biological replicates per treatment) was processed for morphological analysis using a standard hematoxylin‐eosin (HE) staining protocol. Fixed tissues were sequentially dehydrated in a graded ethanol series, cleared in xylene, and embedded in paraffin blocks, from which 6 μm thick sections were obtained using a rotary microtome (RM2235, Leica, Germany). After mounting and baking, sections were stained with HE, followed by dehydration and clearing before being permanently mounted with neutral resin. Microscopic examination was conducted using a research‐grade microscope (ECLIPSE Ni‐U, Nikon, Japan), with subsequent image acquisition and quantitative morphometric analysis performed using ImageJ software. For intestinal morphology assessment, 10 representative measurements each of villus height, muscularis thickness, microvillus length, enterocyte height, and goblet cell density were systematically recorded per section, with the mean values calculated as the final morphological parameters for each sample.

### 2.6. Real‐Time Quantitative PCR Analysis

Total RNA was extracted from liver and intestinal tissues using the RNAsimple Total RNA Kit (TIANGEN, China) and reverse transcribed into cDNA with the PrimeScript RT Reagent Kit (TaKaRa, China). Gene expression was quantified by real‐time PCR on a LightCycler 480 system (Roche, USA) using SYBR Green chemistry with gene‐specific primers (Table 3), following a standardized thermal cycling protocol (95°C for 4 min; 40 cycles of 95°C for 30 s, 60°C for 30 s, and 72°C for 30 s; 72°C for 10 min). Relative expression levels were calculated using the 2^−ΔΔCt^ method with *β-actin* as the reference gene, with all reactions performed in triplicate for reproducibility.

**Table 3:** Real‐time PCR primer sequences.

Target gene	Primer sequence (5′–3′)	Genbank accession number
*β-actin*	F:TACAACGAGCTGAGGGTGGC R:GGCAGGGGTGTTGAAGGTCT	AJ438158.1
*pept1*	F:TGATGACCTGGCCACCTCTA R:ACGACCTGTCCCACCGTATA	KY775396.1
*bckd*	F:TGGACGACCACAAGGACGTG R:AGACGGGAGGTGAGGGTGGT	BX076477
*sd*	F:CAGCCAGGCCTTTGAGTACA R:AAGGTCTCCACTGCTTGCAA	XM_021574025.1
*cpt1*	F:TACAGCTGGCCCAATTCAGG R:TCGCAGTGTTCTTGTCCTCC	AF327058.3
*atgl*	F:GCTGCAGAGTGTTCTTTCGC R:GGGAGTGTTGCAATTCCAGC	KM980090.1
*srebp1*	F:TCCTCTCCCTCAATCCCCTG R:CGAGTCAGCTGCGTTGTCT	KP342261.1
*atgpat*	F:TGCCCAGTGAACCGTAACTC R:CCCTTGCGGTTATTCAGGGT	XM_021623520.1
*fas*	F:TCTAGAGACGCCACCTTCGA R:TGCAGTTTCTCCTCAGCCAG	XM_021581290.1
*glut2*	F:GCCATGACAGTCGGCCTCG R:AATAACTCAGCCACAATGAACCAG	AF246147.1
*gyp*	F:TGATTAACCTGGGGCTGCAG R:GCCATCGAGTCCAGGAAACA	XM_021585435.1
*gk*	F:AGATCACTGTGGGCATCGAC R:GATGTCACAGTGAGGCGTCA	AF053331.2
*pfk*	F:GTGGTGGAGATGCACAAGGA R:GCTTGATGTTGTCCCCTCCA	XM_021579860.1
*pk*	F:GTTCCCTGTCGAGTCTGTGG R:CAGACGACGAAGCTCCTCAA	XM_021622264.1
*gys*	F:GACAGAGAGGCCAACGACTC R:ACTCATGGAAATGGGCGAGG	NM_001281374.1
*fbpase*	F:CCACTGGATGGATCTTCCAACA R:CCCTCTCATTGGGCTCATCG	AF333188.1
*tnf-α*	F:GGCGAGCATACCACTCCTCTGA R:AGCTGGAACACTGCACCAAGGT	AJ401377.1
*il-1β*	F:ACGGTTCGCTTCCTCTTCTACA R:GCTCCAGTGAGGTGCTGATGAA	AJ557021.2
*il-6*	F:CCAGTGAGAGGAAGCGTGTT R:GTCTTTGCCCCTCTTTCCCA	DQ866150.1
*il-8*	F:GTCAGCCAGCCTTGTCGTTGT R:CGTCTGCTTTCCGTCTCAATGC	NM_001124362.1
*il-12α*	F:GAAAGTAGAACCGCCAGCCA R:TGCTCCTCCTTTCCGTTGTC	HE798148.1
*il-12β*	F:ACAGTGATGGTGAAGGCCTG R:AGTGAGTTTGATGCGGGACA	HE798149.1
*il-4r2*	F:CTTGGCAGAGACTTGGAGCC R:CAGTCACTGAAATGGCCGCT	NM_001246336.1
*il-10r1*	F:GTGGACATATGGGACGGGGA R:ATACCTGGCCATCTGCACCT	NM_001281374.1

Abbreviations: *atgl*, adipose triglyceride lipase; *atgpat*, glycerol‐3‐phosphate acyltransferase; *bckd*, branched‐chain alpha‐ketoacid dehydrogenase complex; *β-actin*, beta‐actin; *cpt1*, carnitine palmitoyltransferase 1; *fas*, fatty acid synthase; *fbpase*, fructose‐1,6‐bisphosphatase; *gk*, glucokinase; *glut2*, glucose transporter type 2; *gyp*, glycogen phosphorylase; *gys*, glycogen synthase; *il-10r1*, interleukin 10 receptor subunit alpha 1; *il-12α*, interleukin 12 subunit alpha; *il-12β*, interleukin 12 subunit beta; *il-1β*, interleukin 1 beta; *il-4r2*, interleukin 4 receptor subunit alpha 2; *il-6*, interleukin 6; *il-8*, interleukin 8; *pept1*, peptide transporter 1; *pfk*, phosphofructokinase; *pk*, pyruvate kinase; *sd*, serine dehydratase; *srebp1*, sterol regulatory element‐binding protein 1; *tnf-α*, tumor necrosis factor alpha.

### 2.7. Western Blotting Analysis

Total hepatic proteins were extracted from triploid rainbow trout liver tissues using the Whole Protein Extraction Kit (KGP2500, KeyGEN, China) according to the manufacturer’s protocol, with protein concentration quantified via BCA assay (PC0020, Solarbio, China). Western blot analysis was conducted using specific rabbit primary antibodies (Cell Signaling Technology, USA) targeting total TOR (2983, 1:1000), phospho‐TOR (5536, 1:1000, Ser2448), total S6K1 (9202, 1:1000), and phospho‐S6K1 (9205, 1:1000, Thr389), followed by detection with an anti‐rabbit HRP‐conjugated secondary antibody (7074, 1:10000). Automated immunodetection was performed on the Wes system (WS‐2724, Protein Simple, USA) employing the Wes reagent kit (SM‐W004, Protein Simple, USA), with subsequent quantification of protein phosphorylation levels determined by calculating the grayscale value ratio of phosphorylated protein bands to corresponding TP bands for each biological replicate.

### 2.8. Statistical Analysis

The experimental data were analyzed using SPSS 27.0 (IBM, USA) and expressed as mean ± SE. After verifying normality (Shapiro–Wilk test) and homogeneity of variance (Levene’s test), one‐way ANOVA with Tukey’s HSD post hoc test was applied for parametric data (*p* < 0.05). Orthogonal polynomial contrasts were used to assess linear and quadratic trends among treatment groups, with *α* = 0.05 considered statistically significant.

## 3. Results

### 3.1. Growth Performance and Feed Utilization

As shown in Table 4, no significant differences among dietary treatments were detected for survival, feed conversion ratio, feed intake, or protein efficiency ratio (*p* > 0.05). Final weight and weight gain rate were also not significantly different among treatments (*p* > 0.05), but both variables showed significant linear trends with increasing substitution level (*p* < 0.05). Feed intake did not differ significantly among treatments under the present feeding conditions (*p* > 0.05).

**Table 4:** Effect of fish meal replacement by complex protein on growth performance and feed utilization of triploid rainbow trout fed the experimental diets for 90 days.

Parameters	Diets					*p*‐Value		
	100% FM	25% CP	50% CP	75% CP	100% CP	ANOVA	Linear	Quadratic
Growth performance								
Initial weight (g)	208.1 ± 0.2	208.3 ± 0.1	208.0 ± 0.1	208.4 ± 0.1	208.4 ± 0.2	0.499	0.273	0.573
Final weight (g)	769 ± 14	782 ± 15	799 ± 14	819 ± 6	804 ± 14	0.123	0.022	0.311
Survival rate^1^ (%)	99.2 ± 0.5	99.8 ± 0.3	99.0 ± 0.6	98.8 ± 0.6	99.8 ± 0.3	0.478	0.997	0.405
Weight gain rate^2^ (%)	270 ± 7	275 ± 7	284 ± 6	293 ± 3	286 ± 7	0.130	0.024	0.296
Feed utilization								
Feed conversion ratio^3^	0.93 ± 0.04	0.89 ± 0.01	0.95 ± 0.01	0.90 ± 0.01	0.89 ± 0.01	0.302	0.349	0.667
Feed intake^4^ (%/day)	1.27 ± 0.06	1.23 ± 0.02	1.31 ± 0.02	1.26 ± 0.01	1.25 ± 0.02	0.488	0.921	0.507
Protein efficiency ratio^5^	2.34 ± 0.11	2.43 ± 0.02	2.33 ± 0.03	2.44 ± 0.02	2.46 ± 0.04	0.352	0.160	0.722

*Note:* Values (mean ± standard error) in the same row with different superscripts are significantly different, significance level = 0.05.

^1^Survival rate (SR, %) = 100 × (final amount of fish)/(initial amount of fish),

^2^Weight gain rate (WGR, %) = 100 × [final body weight (g) − initial body weight (g)]/[initial body weight (g)],

^3^Feed conversion ratio (FCR) = feed fed (g)/body weight gain (g),

^4^Feed intake (FI, %/day) = 100 × feed fed (g)/[days × (initial body weight (g) + final body weight (g))/2],

^5^Protein efficiency ratio (PER) = body weight gain (g)/[fish fed (g) × dietary protein level (%)].

### 3.2. Body Indices and Compositions

As shown in Table 5, condition factor (CF) increased linearly with substitution level (*p* < 0.05), and the 75% CP and 100% CP groups showed significantly higher values than the 100% FM group. Intestinal length index (ILI) and whole‐body crude protein content were significantly affected by dietary treatment (*p* < 0.05), with significant linear and quadratic responses. The 100% FM group showed lower ILI values than the substituted groups, and whole‐body crude protein content was higher in the 100% CP group than in the 100% FM group. No significant differences were detected in viscerosomatic index, hepatosomatic index, moisture, crude lipid, or ash content (*p* > 0.05).

**Table 5:** Effect of fish meal replacement by complex protein on body indices and compositions of triploid rainbow trout fed the experimental diets for 90 days.

Parameters	Diets					*p*‐Value		
	100% FM	25% CP	50% CP	75% CP	100% CP	ANOVA	Linear	Quadratic
Body indices								
Condition factor^1^	1.76 ± 0.03^a^	1.82 ± 0.03^ab^	1.82 ± 0.02^ab^	1.90 ± 0.02^b^	1.89 ± 0.02^b^	<0.001	<0.001	0.425
Viscerosomatic index (%)^2^	10.8 ± 0.4	10.8 ± 0.4	10.9 ± 0.3	10.0 ± 0.3	10.8 ± 0.3	0.344	0.572	0.652
Hepatosomatic index (%)^3^	1.14 ± 0.03	1.15 ± 0.03	1.12 ± 0.03	1.12 ± 0.04	1.17 ± 0.02	0.740	0.842	0.291
Intestinal length index^4^	0.331 ± 0.010^a^	0.379 ± 0.007^b^	0.383 ± 0.011^b^	0.381 ± 0.005^b^	0.380 ± 0.010^b^	<0.001	0.001	0.004
Body compositions								
Moisture (%)	66.0 ± 0.3	65.3 ± 0.2	65.1 ± 0.6	64.8 ± 0.2	65.3 ± 0.6	0.356	0.175	0.144
Crude protein (% wet basis)	17.0 ± 0.3^ab^	16.9 ± 0.2^ab^	16.7 ± 0.1^a^	17.6 ± 0.3^bc^	18.2 ± 0.2^c^	<0.001	<0.001	0.004
Crude lipid (% wet basis)	14.0 ± 0.3	14.2 ± 0.4	15.0 ± 0.6	14.4 ± 0.6	14.6 ± 0.7	0.719	0.465	0.541
Ash (% wet basis)	1.58 ± 0.06	1.68 ± 0.08	1.76 ± 0.11	1.79 ± 0.06	1.61 ± 0.07	0.373	0.468	0.069

*Note:* Values (mean ± standard error) in the same row with different superscripts are significantly different, significance level = 0.05.

^1^Condition factor (CF) = 100 × [body weight (g)]/[body length (cm)]^3^,

^2^Viscerosomatic index (VSI, %) = 100 × [viscera weight (g)]/[body weight (g)],

^3^Hepatosomatic index (HSI, %) = 100 × [hepatic weight (g)]/[body weight (g)],

^4^Intestinal length index (ILI) = intestine length (mm)/body length (mm).

### 3.3. Protein Digestion and Metabolism

**Figure 1:**
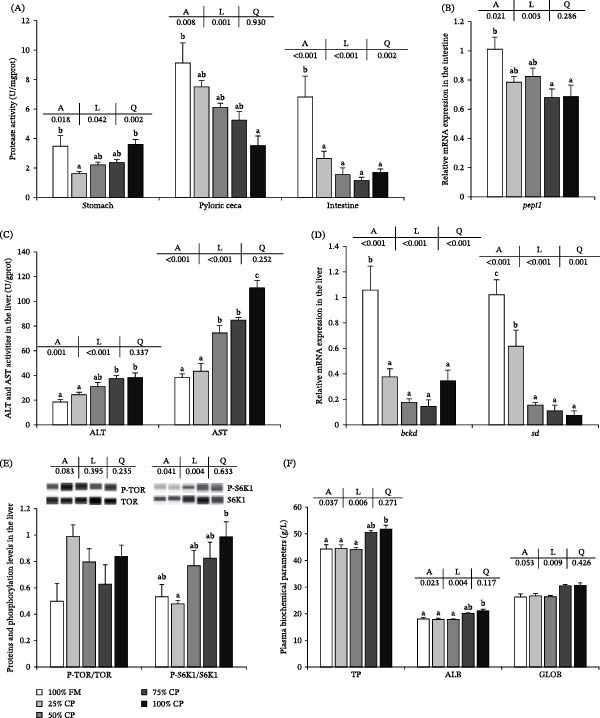
Effect of fish meal replacement by complex protein on protein digestion and metabolism of triploid rainbow trout fed the experimental diets for 90 days. Bars with different superscripts are significantly different (A: ANOVA, L: linear trend, Q: quadratic trend; significance level = 0.05). (A) Protease activity of stomach, pyloric ceca, and intestine. (B) Expression of protein absorption‐related mRNA in intestine. (C) Activity of amino acid metabolizing enzymes in liver. (D) Expression of mRNA related to amino acid metabolism in liver. (E) Proteins and phosphorylation levels related to protein synthesis in liver. (F) Plasma biochemical parameters. ALB, albumin; ALT, alanine aminotransferase; AST, aspartate aminotransferase; GLOB, globulin; TP, total protein.

The protein digestion‐ and metabolism‐related parameters are shown in Figure 1. Stomach protease activity was significantly affected by dietary treatment and showed a quadratic response to substitution level (*p* < 0.05; Figure 1A). Protease activity in the pyloric ceca and intestine decreased linearly with increasing substitution level (*p* < 0.05). Intestinal *pept1* mRNA expression also decreased linearly with increasing substitution level (*p* < 0.05; Figure 1B).

In the liver, ALT and AST activities increased with increasing substitution level (*p* < 0.05; Figure 1C). The mRNA expression levels of *bckd* and *sd* were significantly affected by dietary treatment and showed linear and quadratic decreases (*p* < 0.05; Figure 1D).

For protein phosphorylation‐related indices in the liver (Figure 1E), S6K1 phosphorylation increased linearly with substitution level (*p* < 0.05), whereas TOR phosphorylation did not differ significantly among treatments (*p* > 0.05). Plasma TP and ALB levels were significantly higher in the 100% CP group than in the 100% FM, 25% CP, and 50% CP groups (*p* < 0.05; Figure 1F), whereas GLOB was not significantly affected by dietary treatment (*p* > 0.05). TP, ALB, and GLOB all showed significant linear responses to increasing complex protein level (*p* < 0.05).

### 3.4. Lipid Digestion and Metabolism

**Figure 2:**
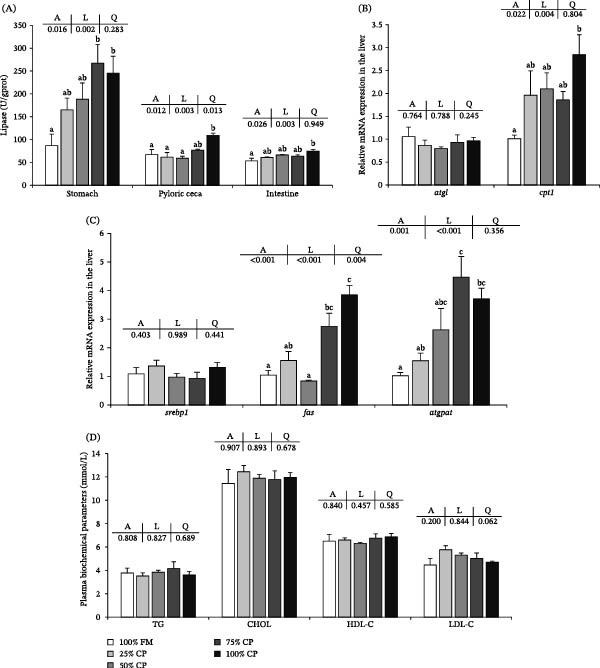
Effect of fish meal replacement by complex protein on lipid digestion and metabolism of triploid rainbow trout fed the experimental diets for 90 days. Bars with different superscripts are significantly different (A: ANOVA, L: linear trend, Q: quadratic trend; significance level = 0.05). (A) Lipase activity of stomach, pyloric ceca, and intestine. (B) Expression of mRNA related to lipid catabolism in liver. (C) Expression of mRNA related to lipid anabolism in liver. (D) Plasma biochemical parameters. CHOL, cholesterol; HDL‐C, high‐density lipoprotein cholesterol; LDL‐C, low‐density lipoprotein cholesterol; TG, triglyceride.

The lipid digestion and metabolism‐related parameters are shown in Figure 2. Lipase activity in the stomach, pyloric ceca, and intestine increased linearly with substitution level (*p* < 0.05; Figure 2A). In the stomach, lipase activity was higher at substitution levels of 75% and 100% than in the control group (*p* < 0.05), whereas in the pyloric ceca and intestine, significantly higher values were observed only in the 100% CP group (*p* < 0.05).

In the liver, *cpt1* and *atgpat* mRNA expression levels increased linearly with substitution level (*p* < 0.05; Figure 2B,C). *fas* mRNA expression showed significant linear and quadratic responses, with higher values in the 75% CP and 100% CP groups (*p* < 0.05). No significant differences were detected in *atgl* or *srebp1* mRNA expression among treatments (*p* > 0.05). Plasma TG, CHOL, HDL‐C, and LDL‐C were not significantly affected by dietary treatment (*p* > 0.05; Figure 2D).

### 3.5. Carbohydrate Digestion and Metabolism

**Figure 3:**
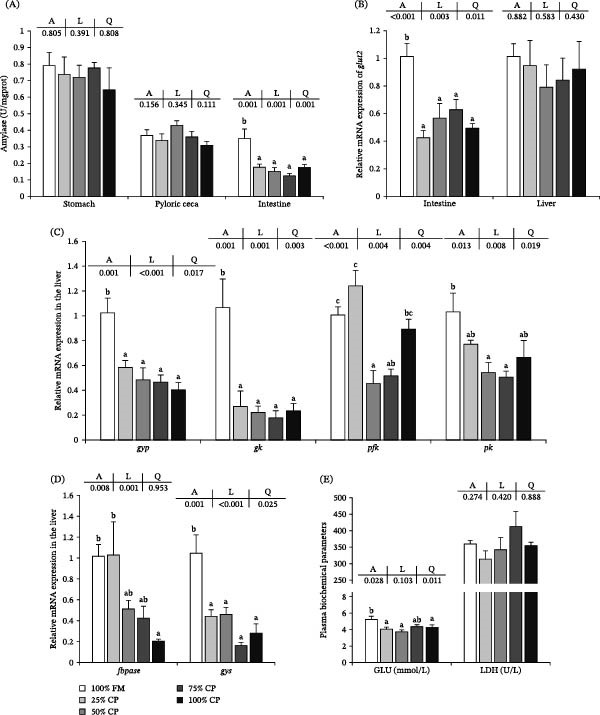
Effect of fish meal replacement by complex protein on carbohydrate digestion and metabolism of triploid rainbow trout fed the experimental diets for 90 days. Bars with different superscripts are significantly different (A: ANOVA, L: linear trend, Q: quadratic trend; significance level = 0.05). (A) Amylase activity of stomach, pyloric ceca, and intestine. (B) Expression of mRNA related to carbohydrate absorption in intestine and liver. (C) Expression of mRNA related to carbohydrate catabolism in liver. (D) Expression of mRNA related to carbohydrate anabolism in liver. (E) Plasma biochemical parameters. GLU, glucose; LDH, lactate dehydrogenase.

The carbohydrate digestion‐ and metabolism‐related parameters are shown in Figure 3. Amylase activity in the stomach and pyloric ceca was not significantly affected by dietary treatment (*p* > 0.05), whereas intestinal amylase activity showed significant linear and quadratic decreases with increasing substitution level (*p* < 0.05; Figure 3A).

Intestinal *glut2* mRNA expression showed significant linear and quadratic decreases with increasing substitution level (*p* < 0.05), whereas hepatic glut2 expression did not differ significantly among treatments (*p* > 0.05; Figure 3B). In the liver, the mRNA expression levels of *gyp*, *gk*, *gys*, *pfk*, *pk*, and *fbpase* were significantly affected by dietary treatment (*p* < 0.05; Figure 3C,D). *gyp*, *gk*, and *gys* differed significantly between the 25% CP group and the control group, whereas *fbpase* showed a significant linear response and differed significantly in the 100% CP group relative to the 100% FM and 25% CP groups. *pfk* and *pk* showed quadratic responses, with an initial decrease followed by a slight increase at higher substitution levels. Plasma GLU concentration showed a significant quadratic decrease with increasing substitution level (*p* < 0.05), whereas LDH activity did not differ significantly among treatments (*p* > 0.05; Figure 3E).

### 3.6. Liver Health

**Figure 4:**
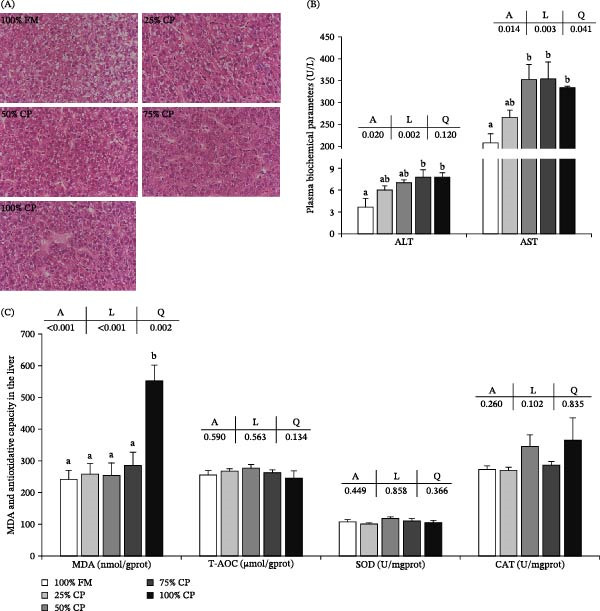
Effect of fish meal replacement by complex protein in the liver health of triploid rainbow trout fed the experimental diets for 90 days. Bars with different superscripts are significantly different (A: ANOVA, L: linear trend, Q: quadratic trend; significance level = 0.05). (A) Histological appearance of liver (hematoxylin‐eosin staining; magnification × 400; scale plate: 50 μm). (B) Plasma biochemical parameters. (C) antioxidative capacity in liver. ALT, alanine aminotransferase; AST, aspartate aminotransferase; CAT, catalase; MDA, malondialdehyde; SOD, superoxide dismutase; T‐AOC, total antioxidative capacity.

The liver‐related parameters are shown in Figure 4. Histological examination did not show marked structural differences among treatment groups under the conditions of the present study (Figure 4A).

In plasma, ALT activity increased linearly with substitution level (*p* < 0.05), and the 75% CP and 100% CP groups showed significantly higher values than the control group (Figure 4B). AST activity showed significant linear and quadratic responses (*p* < 0.05), with higher values in the groups receiving 50% substitution or above than in the control group.

In the liver, MDA content was significantly affected by dietary treatment and showed linear and quadratic responses, with the highest value observed in the 100% CP group (*p* < 0.05; Figure 4C). No significant differences were detected in T‐AOC, SOD, or CAT activities among treatments (*p* > 0.05).

### 3.7. Intestine Health

The intestine‐related parameters are shown in Figure 5. Villus height increased linearly with substitution level (*p* < 0.05), whereas enterocyte height decreased linearly (*p* < 0.05). Goblet cell density showed a significant quadratic response, with higher values at 25% CP and 50% CP than at the highest substitution level (*p* < 0.05; Figure 5A1,A2,B). No significant differences were detected in muscular layer thickness or microvillus height (*p* > 0.05).

Figure 5Effect of fish meal replacement by complex protein in the intestine health of triploid rainbow trout fed the experimental diets for 90 days. Bars with different superscripts are significantly different (A: ANOVA, L: linear trend, Q: quadratic trend; significance level = 0.05). (A) Histological appearance of intestine (hematoxylin‐eosin staining; a: villus height, b: muscular layer thickness, c: enterocyte height, d: microvillus, e: goblet cell; A1: magnification × 100, scale plate: 100 μm; A2: magnification × 400, scale plate: 50 μm). (B) Intestine morphological parameters. (C) Antioxidative capacity in intestine. (D1, D2) Expression of mRNA related to proinflammatory cytokine in intestine. (E) Expression of mRNA related to anti‐inflammatory cytokine in intestine. CAT, catalase; GSH‐PX, glutathione peroxidase; MDA, malondialdehyde; SOD, superoxide dismutase; T‐AOC, total antioxidative capacity.

**Figure:**
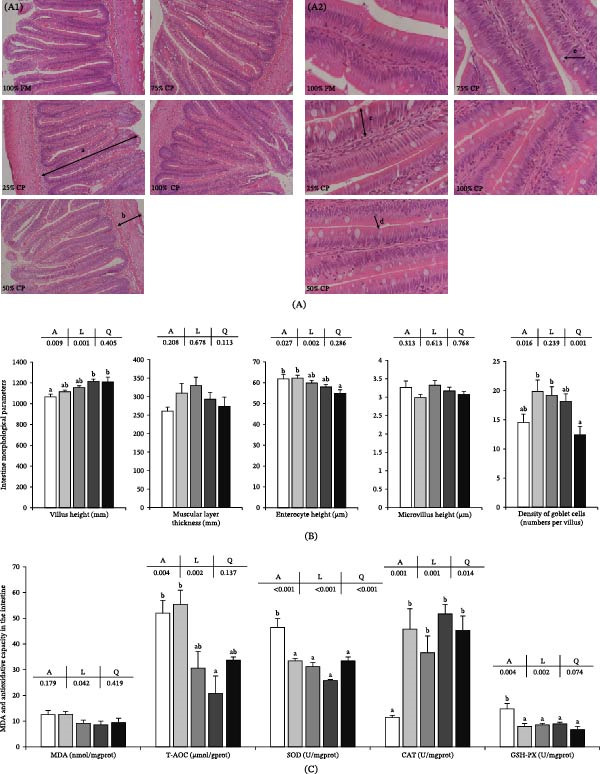

**Figure:**
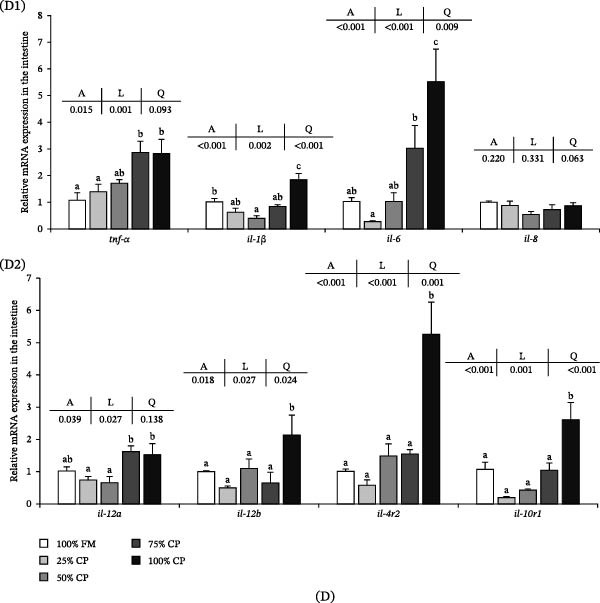

For intestinal oxidative indices (Figure 5C), MDA content and T‐AOC showed quadratic responses to substitution level (*p* < 0.05). T‐AOC reached its lowest value in the 75% CP group. SOD and GSH‐PX activities decreased significantly with dietary substitution (*p* < 0.05), whereas CAT activity increased significantly (*p* < 0.05).

The intestinal cytokine‐related mRNA expression data are shown in Figure 5D1,D2,E. *tnf-α* and *il-12α* expression levels increased linearly with substitution level (*p* < 0.05), and the 75% CP and 100% CP groups showed significantly higher values than the control group. *il-8* expression was not significantly affected by dietary treatment (*p* > 0.05). The mRNA expression levels of *il-1β*, *il-6*, *il-12β*, *il-4*, *il-4r2*, and *il-10r1* showed significant linear or quadratic responses (*p* < 0.05), with lower values at 25% CP or 50% CP and higher values in the 100% CP group.

## 4. Discussion

In the present study, complete replacement of FM with the complex protein blend did not significantly impair growth performance or feed utilization in triploid rainbow trout during the 12‐week trial, although final weight and weight gain rate showed significant linear trends with increasing substitution level. However, these apparently positive outcomes should be interpreted cautiously, because high replacement levels were also accompanied by elevated ALT and AST activities, increased hepatic MDA content, and linear upregulation of intestinal *tnf-α* and *il-12α*. Therefore, the present results indicate that growth was maintained together with measurable physiological cost, rather than demonstrating that complete FM replacement was fully safe or physiologically neutral. Because this trial was conducted in subadult triploid rainbow trout, the findings should be interpreted within that specific life stage and ploidy context [31, 32]. Responses at high replacement levels can differ in juvenile trout [26, 27].

Previous studies in rainbow trout indicate that the response to high FM replacement is strongly formulation‐ and ingredient‐dependent. In juvenile trout, a mixture of animal byproducts supported only limited FM replacement without adverse growth [27]. Ingredient evaluation studies have also shown marked differences among soybean, pea, and canola meals and their concentrates [29]. By contrast, optimized FM‐free plant‐protein combinations supplemented with essential amino acids, taurine, and krill‐based attractants maintained growth in larger rainbow trout [19], whereas blend‐based replacement in juvenile trout supported acceptable growth at 63%–82% replacement but not always at 100% replacement [26]. Accordingly, the present results are better interpreted as evidence of short‐term growth maintenance under this specific nutritionally compensated complex‐protein formulation, rather than as proof that complete FM replacement is broadly successful or risk‐free in rainbow trout [19, 26, 27, 29].

Another point that should be interpreted cautiously is feed acceptance of the 100% CP diet. Although feed intake did not differ significantly among treatments, the unchanged FI values should not be overinterpreted as evidence that the palatability of the 100% CP diet was fully equivalent to that of the FM‐based control. In rainbow trout, optimized FM‐free plant‐protein diets have used krill products specifically as feed attractants to avoid feed intake depression [19]. By contrast, the present 100% CP diet contained no FM or fish soluble/hydrolysates. Because fish were fed to apparent satiation and FI was estimated at the cage level during a long‐term feeding trial, the present data indicate overall diet acceptance under the tested culture conditions rather than a direct palatability or preference response.

The digestive and protein‐metabolism data further indicate that the complex protein diet altered nutrient utilization patterns in triploid rainbow trout. Increased lipase activity but reduced intestinal amylase activity suggests a shift in digestive priority away from carbohydrate processing, and altered digestive responses have also been reported in fish receiving plant‐protein‐rich diets [35, 36]. At the protein‐metabolism level, reduced intestinal *pept1* expression suggests lower transcription of one pathway for di‐/tripeptide transport [37]. Alternative protein diets can also modify intestinal transport‐related responses and nutrient absorption in fish [36, 38]. However, the lower *pept1* expression observed here does not necessarily contradict the higher plasma TP and ALB and whole‐body crude protein in the high‐substitution group, and is better interpreted as evidence of altered protein handling and allocation rather than reduced overall protein retention.

The downregulation of hepatic *bckd* and *sd* should also be interpreted cautiously. Rather than indicating improved protein utilization efficiency, this pattern may reflect altered amino acid handling and a possible conservation response under relatively imbalanced or limiting amino acid supply. General nutritional studies have shown that an amino acid imbalance can reduce nitrogen retention and increase nitrogen loss [39, 40]. In rainbow trout, long‐term feeding of a plant‐based diet devoid of marine ingredients induced hepatic glutamate dehydrogenase and was associated with lower feed efficiency and protein retention [41]. Therefore, the present data support the conclusion that amino acid metabolism was substantially reorganized under the high‐substitution diets, but they do not allow distinction between improved utilization and compensatory conservation in response to amino acid imbalance or limitation [39–41].

Notably, these metabolic changes occurred together with significantly increased ALT and AST activities and elevated hepatic MDA content, especially in the 100% CP group, indicating clear hepatic stress under complete FM replacement, even in the absence of obvious histological damage during the 12‐week trial. This distinction is important, because biochemical and oxidative indicators can reveal hepatic disturbance before marked structural damage becomes evident [42]. Therefore, maintenance of growth in the 100% CP group should be interpreted as a dissociation between somatic performance and liver health, rather than as evidence that the diet was fully physiologically acceptable [42].

The complex protein diet also modified hepatic lipid metabolism, as reflected by the increased expression of *cpt1*, *fas*, and *atgpat*. However, this pattern should be interpreted conservatively. Fatty acid oxidation and lipogenesis are classically considered reciprocally regulated, and malonyl‐CoA is a well‐established physiological inhibitor of CPT1 [43]. Because the present study measured transcript abundance only and did not determine malonyl‐CoA concentration, CPT1 activity, FAS activity, or net lipid flux, the simultaneous increase in *cpt1* and *fas* mRNA should not be taken as direct evidence that hepatic fatty acid oxidation and de novo lipogenesis were functionally enhanced at the same time. In rainbow trout, diet composition can alter lipid‐sensing and cellular signaling pathways in liver without permitting a simple functional interpretation from transcript responses alone [44]. In addition, because the experimental diet was a multi‐ingredient formulation, the respective contribution of any single dietary component to the observed lipid‐metabolic responses cannot be distinguished.

Carbohydrate metabolism was also affected by complex protein substitution. Reduced intestinal glut2 expression, lower plasma GLU, and downregulation of several hepatic genes involved in glycolysis, gluconeogenesis, and glycogen turnover indicate that carbohydrate handling was modified under the high‐substitution diets. In rainbow trout, macronutrient composition affects TOR‐related signaling and metabolism‐related gene expression [45], and long‐term plant‐based feeding can strongly modify hepatic metabolic enzymes [41]. Similar diet‐dependent changes in intermediary metabolism have also been reported under soybean‐protein replacement in other carnivorous fish [46]. Therefore, maintenance of growth despite reduced intestinal amylase activity and *glut2* expression likely reflects partial metabolic compensation, with greater reliance on lipid utilization and altered amino acid allocation, rather than normal carbohydrate utilization [41, 45, 46].

In addition to the liver response, the intestine was clearly affected by the complex protein diet. Increased villus height, decreased enterocyte height, and changes in goblet cell density indicate structural remodeling of the intestinal epithelium. These changes were accompanied by a clear proinflammatory component, as reflected by the linear upregulation of *tnf-α* and *il-12α* at high replacement levels. In rainbow trout, pea‐protein replacement has been shown to alter digestive tract morphology [47] and to modify growth, health status, gut microbiome, and immune‐ and growth‐related gene expression [48]. Together, these findings support the view that the intestine is one of the main target tissues responding to plant‐protein‐rich replacement strategies [47, 48].

In summary, complete replacement of FM with the complex protein blend maintained short‐term growth performance over 12 weeks in subadult triploid rainbow trout, but this occurred together with clear signs of hepatic stress, oxidative challenge, and intestinal inflammatory activation. From a production perspective, 100% replacement should therefore be regarded as a physiologically risky strategy rather than a fully validated success. Several limitations of the present study should also be emphasized. First, the diet was tested as a combined formulation, so the respective effects of soybean meal, soybean protein concentrate, corn gluten meal, wheat gluten, and blood cell powder could not be separated. Second, only triploid fish were examined, so the present data do not allow direct comparison with diploid trout. Third, no direct palatability assay or attractant comparison was conducted. Fourth, the 12‐week trial may not fully capture delayed deterioration during a longer grow‐out period. Future studies should therefore further dissect the role of individual protein ingredients, compare diploid and triploid trout directly, evaluate palatability and attractant strategies more explicitly, and determine under longer‐term production conditions whether the hepatic and intestinal alterations observed here persist or eventually translate into reduced performance or survival.

## Author Contributions

**Hongdian Zhu**: conceptualization, data curation, formal analysis, methodology, writing – original draft. **Rui Ma**: methodology, writing – review and editing, funding acquisition. **Guoliang Sun and Zezhong Wu**: methodology, writing – review and editing. **Haining Tian**: formal analysis. **Yuqiong Meng**: conceptualization, data curation, funding acquisition, writing – review and editing. **Dong Huang**: conceptualization, data curation, writing – review and editing.

## Funding

This study was funded by Qinghai University Research Ability Enhancement Project (Grant 2025KTSQ19) and the National Natural Science Foundation of China (Grant 31560722).

## Disclosure

An earlier version of this manuscript was previously posted on SSRN: https://ssrn.com/abstract=5370465 or https://doi.org/10.2139/ssrn.5370465.

## Conflicts of Interest

The authors declare no conflicts of interest.

## Data Availability

Data sharing is not applicable to this article, as no datasets were generated or analyzed during the current study.
